# Taking a stab at modelling canine tooth biomechanics in mammalian carnivores with beam theory and finite-element analysis

**DOI:** 10.1098/rsos.220701

**Published:** 2022-10-19

**Authors:** Tahlia I. Pollock, Olga Panagiotopoulou, David P. Hocking, Alistair R. Evans

**Affiliations:** ^1^ School of Biological Sciences, Monash University, Clayton 3800, Australia; ^2^ Monash Biomedicine Discovery Institute, Department of Anatomy & Developmental Biology, Monash University, Clayton 3800, Australia; ^3^ Zoology, Tasmanian Museum and Art Gallery, Hobart, Australia; ^4^ Geosciences, Museums Victoria, Melbourne, Victoria, Australia

**Keywords:** tooth morphology, feeding ecology, biomechanics, Carnivora, form-function

## Abstract

Canine teeth are vital to carnivore feeding ecology, facilitating behaviours related to prey capture and consumption. Forms vary with specific feeding ecologies; however, the biomechanics that drive these relationships have not been comprehensively investigated. Using a combination of beam theory analysis (BTA) and finite-element analysis (FEA) we assessed how aspects of canine shape impact tooth stress, relating this to feeding ecology. The degree of tooth lateral compression influenced tolerance of multidirectional loads, whereby canines with more circular cross-sections experienced similar maximum stresses under pulling and shaking loads, while more ellipsoid canines experienced higher stresses under shaking loads. Robusticity impacted a tooth's ability to tolerate stress and appears to be related to prey materials. Robust canines experience lower stresses and are found in carnivores regularly encountering hard foods. Slender canines experience higher stresses and are associated with carnivores biting into muscle and flesh. Curvature did not correlate with tooth stress; however, it did impact bending during biting. Our simulations help identify scenarios where canine forms are likely to break and pinpoint areas where this breakage may occur. These patterns demonstrate how canine shape relates to tolerating the stresses experienced when killing and feeding, revealing some of the form–function relationships that underpin mammalian carnivore ecologies.

## 1. Introduction

Canine teeth are found in almost all predatory mammals and are used as key tools in prey capture and consumption [1–4]. Carrying out these feeding behaviours places constraints on their shape and, just like other tooth types, their forms vary with specific feeding ecologies [4–7].

The variation of canine shape from robust to slender has been related to the material properties of the prey being handled and the killing behaviour used by a predator [4–6,8]. Robust canines have been correlated with biting ‘hard’ materials (stiff and brittle) like bone or subduing struggling prey, and slender canines with biting ‘soft’ deformable materials (ductile and tough) like muscle or organs [4–6,9,10]. Canine teeth can also be straight or curved [5]. Curved claws and teeth have been associated with prey capture and retention in many animals, including birds, snakes, sharks and seals [11–19]. In predatory mammals, curved canines have been associated with shaking behaviours and are thought to act as hooks at the front of the mouth that prevent the prey from slipping out of grasp [5,13]. Straight canines, on the other hand, have been hypothesized to be advantageous when biting deeply into prey [5]. As canine tooth shape varies, we expect to find differences in how they distribute and tolerate stresses associated with various diets and killing behaviours.

Canines play a central role in carnivore feeding ecology and are used to perform a wide range of behaviours [3,6,20]. They can be used to bite into and kill prey, pull off chunks to consume, or even shake apart prey [1–3,20–22]. These behaviours load canine teeth in distinct ways. When biting into prey, the tip of the tooth makes initial contact with the food and a compressive force is directed along the long axis of the tooth to its base. However, the direction of force will vary depending on the angle that the tooth tip contacts prey [12,19], such as when biting into differently sized prey or biting in distinct ways (encircling the throat versus snapping at flanks). If the tooth is already embedded in the prey, the predator may pull away from the prey or begin shaking it [20,22]. When pulling away, a compressive force is applied to the posterior surface of the tooth, perpendicular to its long axis. If the predator begins shaking prey, a compressive force is applied to the side of the tooth (either lingual or labial surface, depending on the direction of the shake). In addition to the predator pulling and shaking prey, the prey may also pull and shake the predator. For example, when handling struggling prey, which can produce unpredictable loads [4]. We expect that differences in how canine teeth are loaded during feeding will impact the way they distribute and tolerate stress and impact the likelihood of breakage.

Previously, the canine teeth of mammalian carnivores have been modelled as a cantilever using beam theory analysis (BTA) to calculate stress [4,6,8,23–27]. This enabled researchers to relate aspects of canine tooth shape, like lateral compression (whether a canine tooth has a circular versus elliptical cross-section) to strength and show that canines are stronger when pulling (loaded along the anterior–posterior axis), compared to shaking (loaded along the lingual–labial axis) [4]. Van Valkenburgh & Ruff [4] also found that the degree of lateral compression varies among species and is likely related to differences in feeding ecology. For example, species with more circular cross-sections were more likely to be subjected to multidirectional lateral forces, like contacting bone or subduing struggling prey [4]. Similar patterns between canine strength, as determined by BTA, and feeding ecology have been found in other studies [6,24]. BTA has also been applied to model stresses at the tip of canine teeth in an investigation of the evolutionary trade-off between tooth strength and puncture performance [23].

While BTA has advanced our understanding of the fundamental relationships between canine shape and stress, it has limitations. BTA models the tooth as a cylinder, deriving stresses from a single cross-section [8]. It does not consider the full three-dimensional shape of the tooth and is inadequate for modelling important aspects of their shape like robusticity and curvature. Finite-element analysis (FEA) can overcome these limitations, as simulations can model the full three-dimensional structure of a canine tooth as well as constrain and load them in more nuanced and biologically relevant ways [8,28]. FEA has been used to explore this form–function relationship in other pointed structures like teeth of theropods, sharks and snakes, claws of theropods, bird beaks, spider fangs and even wasp and honeybee stingers [15,29–38]. The utility of FEA for modelling canine teeth has already been established in studies estimating breakage loads when pulling and modelling crack propagation and fracture evolution [8,39]. However, the focus of these studies was relatively narrow, investigating a single loading condition in a small sample of teeth or modelling a single tooth. BTA and FEA are the two leading methodologies for the investigation of canine tooth biomechanics, however, no study has directly compared them.

This study investigates how canine tooth forms tolerate stress during feeding. We apply BTA and FEA to model how varying aspects of canine shape impact tooth stress under a range of simulated feeding behaviours and how this relates to diet and killing behaviour. Additionally, we directly compare BTA and FEA and discuss their utility in the analysis of canine tooth biomechanics. To achieve this, we selected and generated canine tooth models from a pre-existing morphospace (see [5]) from geometric morphometrics that span the range of shape variation found in a broad taxonomic sample of mammalian carnivores. These canine teeth were subjected to both BTA and FEA under loading conditions that mimic various biting scenarios, as well as pulling and shaking behaviours.

## 2. Material and methods

### 2.1. Canine tooth sample

Quantification of tooth shape is based on the dataset and shape analysis from Pollock, Hocking [5]. 203 canine teeth representing 63 species (electronic supplementary material, table S1) were sourced from multiple institutions: Museums Victoria (NMV), Australian Museum (AMS), South Australian Museum (SAM), Tasmanian Museum and Art Gallery (TMAG), Smithsonian Institution (USNM), American Museum of Natural History (AMNH), Monash University Zoology Research Collection (MZRC) and State Museum of Natural History Stuttgart (SMNS). Teeth were moulded and cast before scanning with the Laser Design DS-Series 2025 3D scanner and three-dimensional surface meshes were constructed. Additional information on the moulding, casting, scanning and meshing protocols can be found in Pollock, Hocking [5].

### 2.2. Tooth model selection and preparation

**Figure 1.**
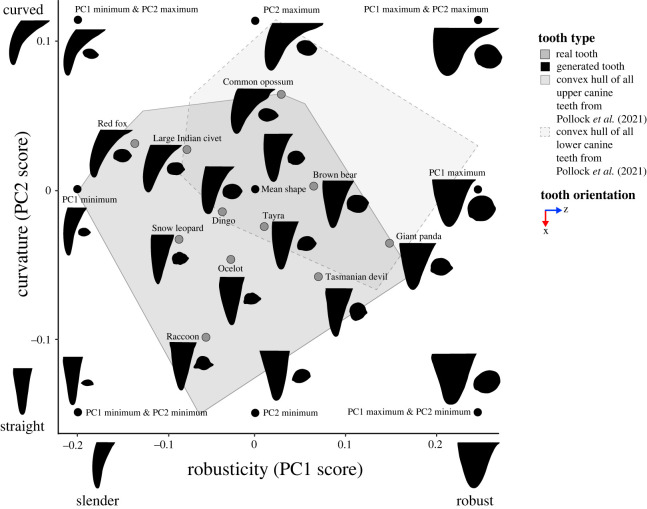
Three-dimensional geometric morphometric (3DGM) morphospace of real and generated tooth models selected for this study showing variation in lateral compression (cross-section taken at 50% tooth height), robusticity (PC1 score) and curvature (PC2 score). The geometric morphometric data and resulting shape space is from Pollock *et al.* [40]. Silhouettes have been scaled to the same tooth height and show teeth in lingual view and the corresponding cross-section taken at 50% tooth height. Convex hulls represent areas of the morphospace occupied by upper and lower canine teeth from Pollock *et al.* [40].

From the morphospace of tooth shapes in Pollock, Hocking [5], we selected 11 teeth that spanned the range of shape variation present (figure 1). Species selected for modelling were giant panda (*Ailuropoda melanoleuca*), brown bear (*Ursus arctos*), Tasmanian devil (*Sarcophilus harrisii*), raccoon (*Procyon lotor)*, ocelot (*Leopardus pardalis*), tayra (*Eira barbara*), dingo (*Canis familiarus dingo*), common opossum (*Didelphis marsupialis*), snow leopard (*Panthera uncia*), large African civet (*Viverra zibetha*) and red fox (*Vulpes vulpes*). For consistency, we selected only upper right canine teeth for all analyses as they cover a majority of the shape space from Pollock, Hocking [5]. Where the upper right tooth was not preserved, the upper left canine was mirror transformed so that it matched the required tooth position. We also generated theoretical canine forms from the morphospace. Tooth meshes of the calculated mean canine shape as well as PC1 and PC2 extremes (nine in total; figure 1) were generated via the warpRefMesh function in geomorph [41], the base tooth mesh chosen for warping was from a dingo (*Canis familiarus dingo*) NMV C22588 (different to the dingo specimen BTA and FEA were applied to). This specimen was selected as it was close to the mean shape of the Pollock, Hocking [5] morphospace.

Including a selection of real canines from species with specific diets and killing techniques allows us to investigate links between canine forms, tooth stress and feeding ecology [4,6]. By contrast, the inclusion of theoretical canine forms enables us to test extreme morphologies that are not found in nature as well as help untangle covariation in aspects of shape, which can limit the investigation of natural forms [42–46]. This combined approach testing real and theoretical canine forms enables us to build a more comprehensive understanding of canine form–function relationships.

The three-dimensional surface mesh files of all tooth models were scaled to the same surface area of 450 mm^2^ (416 mm^2^ was the average of all the surface areas of the unscaled teeth; electronic supplementary material, table S1) before BTA and FEA to enable comparisons of shape and stress independent of size [30,47–49]. This was done in line with Dumont, Grosse [47], who determined that when evaluating stress-strength performance among FE models, size effects can be removed by scaling models to an equal force: surface area ratio.

**Figure 2.**
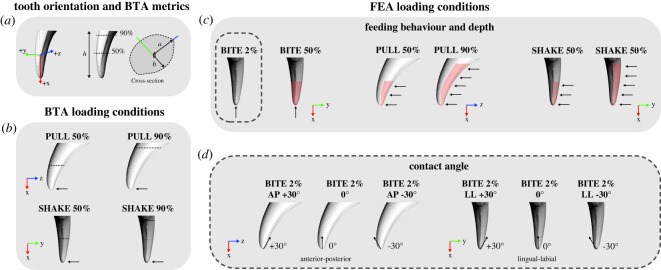
Diagram of tooth orientation, direction of forces and loading conditions applied to tooth models for (*a* and *b*) beam theory analysis (BTA) and (*c* and *d*) finite-element analysis (FEA). Shown on red fox (*Vulpes vulpes*) NMV C25074, where arrows indicate the direction of force applied, dashed lines indicate positions of cross-sections for BTA, and red shaded areas indicate surfaces where force was applied for FEA. (*a*) Canine tooth orientation in three-dimensional space for all analyses (anterior–labial view) and metrics used to calculate stress via BTA, where *h* is tooth height, *a* is half of the canine tooth width along the anterior-posterior axis, and *b* is half of the canine tooth width along the lingual–labial axis (as per Van Valkenburgh and Ruff (4)). (*b*) BTA simulating pulling and shaking feeding behaviours: PULL 50%, PULL 90%, SHAKE 50% and SHAKE 90% loading conditions. (*c*) FEA simulating biting, pulling and shaking feeding behaviours: BITE 2%, BITE 50%, PULL 50%, PULL 90%, SHAKE 50% and SHAKE 90% loading conditions. (*d*) FEA simulating varying contact angles between tooth and prey: BITE 2% AP +30°, BITE 2% AP −30°, BITE 2% LL +30°, BITE 2% LL −30°. Loading cases are static. Terms BITE, PULL and SHAKE are used to describe the simulated static loads.

Each tooth model was aligned to a global coordinate system in Rhinoceros 5 (McNeel, North America, USA) so that the dorsal–ventral axis of the tip of the tooth was parallel to the *x*-axis, lingual–labial axis parallel to the *y*-axis, and the anterior–posterior axis parallel to the *z*-axis in three-dimensional space (figure 2*a*).

### 2.3. Measures of canine tooth shape

We measured the degree of lateral compression for each scaled and oriented tooth model as a ratio of anterior–posterior (AP) width/lingual–labial (LL) width:

$$\mathrm{Lateral}\;\mathrm{compression}\;=\frac{\mathrm{AP}\;\mathrm{width}\;(\mathrm{mm})}{\mathrm{LL}\;\mathrm{width}\;(\mathrm{mm})}$$

Anterior–posterior width and lingual–labial width were measured in Rhinoceros 5, from segments of the canine made perpendicular to the midline of the tooth (figure 2*a*). Segments were taken at 90% of the tooth height and 50% of the tooth height (figure 2*a*). We measured tooth height as the maximum height of the tooth along the superior–inferior *x* axis, from the tip of the tooth to the highest point of the basal boundary of the enamel as per Pollock, Hocking [5].

Canine robusticity and curvature were measured previously via three-dimensional geometric morphometrics in Pollock, Hocking [5]. Canine tooth landmarks were collected, subjected to a Generalized Procrustes Analysis (GPA), and a Principal Components Analysis (PCA) to produce a morphospace of canine tooth shape (see fig. 3 in [5]). In this morphospace, PC2 correlates with curvature and PC1 primarily correlates with robusticity; however, there is also some degree of lateral compression captured in PC1 (figure 1). The overall relative contribution of lateral compression to PC1 score was determined to be negligible (see electronic supplementary material section 1.1 and figure S1). Hence, in this study we used PC1 score as a proxy for robusticity, PC2 score as a proxy for curvature, and assessed lateral compression using the abovementioned metric.

**Figure 3.**
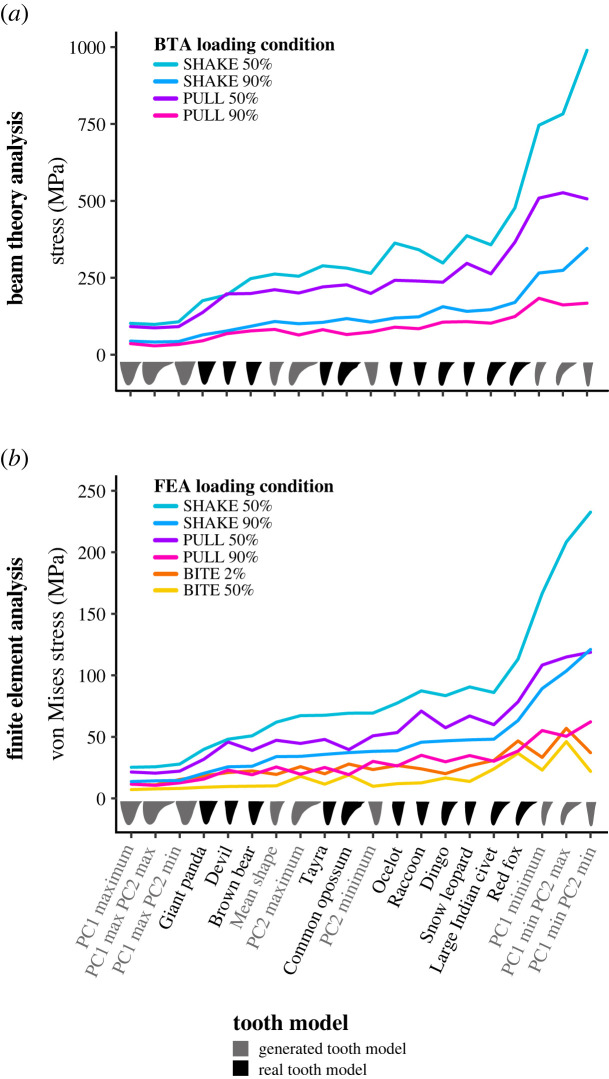
Calculated maximum stresses from (*a*) beam theory analysis (BTA) and (*b*) finite-element analysis (FEA) simulations for each tooth model under loading conditions: BITE 2% and 50%, PULL 50% and 90% and SHAKE 50% and 90%. FEA maximum stress value for each tooth model is the 90^th^ percentile of von Mises stress values for all surface nodes. Force applied was 300 N for BTA and FEA under all loading conditions. Tooth models ordered for increasing FEA SHAKE 50% stress.

### 2.4. Beam theory analysis (BTA)

#### 2.4.1. Loading conditions and maximum stress calculations

As per Van Valkenburgh & Ruff [4] we applied BTA to simulate unidirectional pulling and shaking feeding behaviours in canine teeth. To simulate pulling (PULL), force was applied in the anterior direction (negative *z* axis), and to simulate shaking (SHAKE), force was applied in the lingual direction (negative *y* axis) of the tooth (figure 2*b*). While our loading conditions are approximations of the feeding behaviours due to being static and unidirectional simulations, we have used the terms PULL and SHAKE for consistency with previous studies [30,32,48–52]. We chose to simulate these behaviours at 50% and 90% of the tooth height to vary the amount of tooth embedded in the prey giving four loading conditions (PULL 50%, PULL 90%, SHAKE 50% and SHAKE 90%). Then we calculated the maximum stresses under each loading condition as per Van Valkenburgh & Ruff [4] according to the equations:

$${\mathrm{SM}}_{\mathrm{LL}}=\;\frac{\pi ba^{3}}{4}$$

and

$${\mathrm{SM}}_{\mathrm{AP}}=\;\frac{\pi ab^{3}}{4}\;$$

where *a* is half the anterior–posterior diameter and *b* is half the lingual–labial diameter, SM_LL_ is equal to the second-moment area about the lingual–labial axis (PULL) and SM_AP_ is equal to the second-moment area about the anterior–posterior axis (SHAKE).

$$\mathrm{Max}\;\mathrm{stress}\;\mathrm{when}\;\mathrm{loaded}\;\mathrm{along}\;\mathrm{the}\;\mathrm{AP}\;\mathrm{axis}\;\;(\mathrm{pulling})=\frac{Fha}{{\mathrm{SM}}_{\mathrm{LL}}}$$

and

$$\mathrm{Max}\;\mathrm{stress}\;\mathrm{when}\;\mathrm{loaded}\;\mathrm{along}\;\mathrm{the}\;\mathrm{LL}\;\mathrm{axis}\;(\mathrm{shaking})=\frac{Fhb}{{\mathrm{SM}}_{\mathrm{AP}}}\;$$

where *F* is force and *h* is the height of the tooth.

Due to the theoretical nature of their study, Van Valkenburgh & Ruff [4] did not vary the force (N) applied when calculating maximum stress (setting *F* = 1); here we included a force of 300 N (*F* = 300). There is no *in vivo* bite force data measured at the canine teeth of carnivorans. The value of 300 N value was chosen based on estimates of bite force generated at the canine teeth of carnivorans (*Canis familiaris* = 351.5 N) and *in vivo* pull force data for the Komodo dragon (maximum values of between 200 and 250 N) [53,54]. As this study aims to test how tooth morphology affects stress among species and loading conditions, non-physiological loads do not impact model comparisons [29], just absolute stress values but in a similar manner for each tooth. Moreover, for consistency we applied the same force (*F* = 300 N) across all loading conditions. See electronic supplementary material, table S2 for BTA measurements and maximum stress calculations for all loading conditions.

#### 2.4.2. Analysis

All data manipulation and analysis were performed using RStudio statistical and graphical environment (R v. 4.0.3, RStudio v. 1.3.1093) [55]. To test for correlations between aspects of canine tooth shape (lateral compression, robusticity and curvature) and the BTA stress of tooth models we performed a series of linear regressions. First, we regressed the lateral compression metric against the BTA stress for specific loading conditions. Second, we performed the same analyses for robusticity and curvature, where we regressed either PC1 score (robusticity) or PC2 score (curvature) against the BTA stress for specific loading conditions.

### 2.5. Finite-element analysis (FEA)

#### 2.5.1. Model creation

All tooth surface meshes were converted into volumetric mesh files of solid (continuum) linear tetrahedral elements (C3D4) of 0.2 mm size (electronic supplementary material, table S3) in 3-Matic v. 14.0 (Materialise, Leuven, Belgium) and exported to Abaqus CAE Simulia 2019 (Dassault Systémes, Velizy-Villacoublay) for FEA.

All teeth were assigned isotropic, homogeneous and linear elastic material properties values from the literature: Young's modulus (*E* = 18 000 MPa) and Poisson's ratio (*ν* = 0.31) from available data of human dentine [33,56]. As our study investigates the response of the overall tooth shape to loading conditions simulating biting, pulling, and shaking, not the influence of microstructures in stress dissipation, we gave the teeth uniform material properties of dentine [29,33] and did not model the external enamel, internal dentine or pulp cavity layers [29,48]. In addition to assuming volumes of enamel, dentine and pulp cavity are consistent among species.

#### 2.5.2. Loads and constraints

Our loading conditions mimic a range of unidirectional biting scenarios, as well as pulling and shaking. For consistency with BTA, a compressive force of 300 N was applied across all loading conditions. Again, although our loading conditions are approximations of the feeding behaviours, we have used the terms BITE, PULL and SHAKE for consistency with previous studies [30,32,48–52].

To simulate biting (BITE) we applied a compressive force to surface nodes at the tip of the tooth, along the long axis (dorsal direction). To simulate pulling (PULL) where part of the tooth is already embedded in the prey, we applied a compressive force to the nodes at the posterior surface of the tooth, in the anterior direction. To simulate shaking (SHAKE), where part of the tooth is already imbedded in the prey, we applied a compressive force to the nodes at the labial surface of the tooth, in the lingual direction (perpendicular to PULL) (figure 2*c*).

Within each of our loading conditions, we varied the surface area of the tooth that was loaded. For load BITE we applied two conditions, where the tip of the tooth was either loaded to 2% of the tooth height, to simulate initial biting (BITE 2%) or 50% of the tooth height, to simulate biting when more of the tooth is embedded in the prey (BITE 50%) (figure 2*c*). We applied two conditions for load PULL, where the posterior surface of the tooth was either loaded to 50% of the tooth height (PULL 50%) or 90% of the tooth height (PULL 90%), to vary the amount of tooth embedded in the prey (figure 2*c*). We did the same for load SHAKE, but varied the labial surface loaded (SHAKE 50% and SHAKE 90%) (figure 2*c*). The number of nodes selected varied among models within each loading condition such that the total of all loads on the nodes was 300 N. All teeth were fixed at their base, by selecting all nodes at the dorsal surface of the tooth and constraining translation (U1, U2, U3) and rotation (UR1, UR2, UR3).

The contact angle between the tooth and the prey remained the same for almost all loading conditions so that the tip was perpendicular to the food (0°). However, for initial biting simulations (BITE 2%) contact angles were varied to simulate different ‘off-angle’ (where the tip of the tooth is not perpendicular to the food) biting scenarios (figure 2*d*). Contact angles varied in the anterior–posterior direction (*x*–*z* plane) so that the tip of the tooth was loaded at a −30° angle (BITE 2% AP −30°) and at a +30° angle (BITE 2% AP +30°). In the lingual–labial direction (*x*–*y* plane) contact angles were varied so that the tip of the tooth was loaded at a −30° angle (BITE 2% LL +30°) and at a +30° angle (BITE 2% LL +30°). See electronic supplementary material, table S3 for additional information on the loads applied for FEA and electronic supplementary material, table S4 for vector calculations for off-angle biting simulations.

#### 2.5.3. Model solution and analysis

All finite-element models were solved using the default implicit direct static solver in Abaqus. Von Mises stress distributions for models and loading conditions were examined qualitatively using colour maps, where colours represent stress magnitudes. Combined morphospaces were generated with 3DGM data (from [5]) and von Mises stress distributions for a selection of loading conditions (BITE 2%, PULL 50% and SHAKE 50%) to look for relationships between canine shape and stress. Deformation regimes among models were assessed using static images of the undeformed model with the deformed model superimposed.

Single summary values of von Mises stress or strains, like average von Mises stress [29,32,48,49] or strains [50] and maximum von Mises stress [29,52] or strains [51] and median von Mises stress [57] have been used in previous research as indicators of biomechanical performance in a comparative context [48], or to undertake subsequent statistical analyses [15,32,51]. While such numerical comparisons defeat the primary purpose of FEA, modelling the distribution of stresses and strain across a complex structure, they can still yield valuable insights in a simplified comparative context. We calculated the ‘maximum’ von Mises stress value (analogous to the BTA calculated maximum stress value) for each tooth model under all loading conditions to permit direct comparison between our BTA and FEA results. This was done by exporting the von Mises stress values for all surface nodes and calculating the 90^th^ percentile (determined to be most representative of the ‘maximum’ stress via sensitivity analysis of: full dataset, 99th percentile, 95th percentile or 90th percentile (electronic supplementary material, figure S2) to avoid the influence of individual stress singularities at constrained or loaded nodes [48,51,52]). In all places in this study, ‘maximum’ refers to the 90^th^ percentile of von Mises stress for FEA.

We used this maximum von Mises stress value to test for correlations between aspects of canine shape and FEA von Mises stress in a similar manner to our BTA (section 2.5.3). We regressed the lateral compression metric against the FEA maximum von Mises stress for all loading conditions. We performed the same analyses for robusticity and curvature, where we regressed either PC1 score (robusticity) or PC2 score (curvature) against the FEA maximum von Mises stress for all loading conditions.

### 2.6. Canine shape, stress and feeding ecology in a comparative context

To directly relate canine shape, tooth stress and feeding ecology to one another we incorporated diet and killing technique categories from Pollock, Hocking [5] as reference for all tooth shapes. Diet categories are based on the range of material properties present in the types of prey consumed, and killing technique categories were based on the documented observations of killing behaviour(s) used by mammalian predators (see Pollock, Hocking [5] for more detail). Categories were overlaid as convex hulls in the combined 3DGM and von Mises morphospaces for a selection of loading conditions (BITE 2%, PULL 50%, SHAKE 50%). This allowed for the investigation of broader patterns.

### 2.7. Comparison of BTA and FEA models

To directly compare BTA stresses and the maximum von Mises stress values from our FEA simulations, we undertook a series of linear regressions. We regressed the BTA stresses of all tooth models against their von Mises stresses under PULL and SHAKE loading conditions (as BITE was not simulated via BTA it was not included in this comparison).

## 3. Results

### 3.1. Beam theory analysis (BTA)

#### 3.1.1. Tooth stress among loading conditions

Our BTA shows that when loading a tooth over a larger surface area (e.g. PULL 50% versus PULL 90% or SHAKE 50% versus SHAKE 90%), the tooth with the smaller loaded area experienced higher stresses (figure 3*a*). We also found a general pattern for each tooth model among loading conditions, where the highest stresses were observed under shaking loads followed by pulling loads (red fox: SHAKE 50% = 477.18 MPa and PULL 50% = 365.70 MPa). However, in a few instances (Tasmanian devil, PC1 max, PC1 max & PC2 min and PC1 max & PC2 max) similar stresses were found for PULL 50% and SHAKE 50% loading conditions and for PULL 90% and SHAKE 90% (figure 3*a*; electronic supplementary material, figure S3).

#### 3.1.2. Tooth stress among models

Of the real tooth models tested, the highest BTA stresses were observed in the red fox under all loading conditions (PULL 50% = 365.07 MPa, PULL 90% = 124.07 MPa, SHAKE 50% = 477.18 MPa, SHAKE 90% = 169.94 MPa) (figure 3*a*). The large Indian civet, snow leopard, ocelot and raccoon also experienced high stresses; however, these varied among loading conditions. The lowest BTA stresses were observed in the giant panda under all loading conditions (PULL 50% = 137.16 MPa, PULL 90% = 45.20 MPa, SHAKE 50% = 175.54 MPa, SHAKE 90% = 64.41 MPa). The Tasmanian devil and brown bear also experienced low stresses (figure 3*a*).

For tooth models generated via geometric morphometrics, high BTA stresses were observed in models generated from PC1 extremes (PC1 min, PC1 min & PC2 max and PC1 min & PC2 min) (figure 3*a*). Again, the models with the highest stresses varied among loading conditions. Low stresses were also observed in tooth models generated from PC1 extremes (PC1 max, PC1 max & PC2 min and PC1 max & PC2 max), and was relatively consistent among loading conditions. Curved and straight tooth models (PC2 min & PC2 max) experienced stresses that fell in the mid-range of the other models for all loading conditions (figure 3*a*).

#### 3.1.3. Tooth stress and canine shape

##### 3.1.3.1. Lateral compression

**Figure 4.**
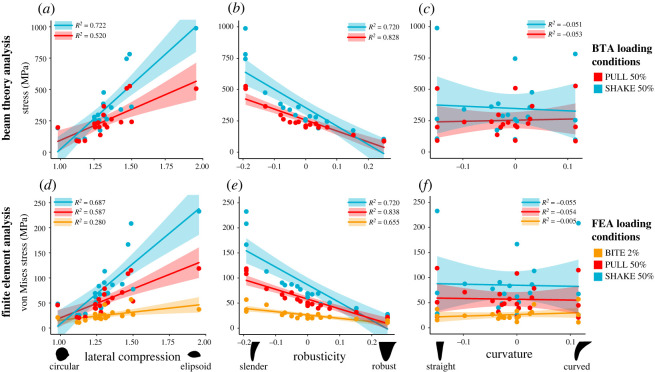
Modelling canine teeth with beam theory (BTA) and finite-element analysis (FEA): relationships between BTA or FEA maximum stress and aspects of canine tooth shape under loading conditions BITE 2%, PULL 50% and SHAKE 50%. Showing (*a* and *d*) lateral compression (anterior–posterior width / lingual–labial width measured at 50% of the tooth height), (*b* and *e*) robusticity (PC1 score) and (*c* and *f*) curvature (PC2 score). FEA maximum stress value for each tooth model is the 90th percentile of von Mises stress values for all surface nodes. Force applied was 300 N for BTA and FEA under all loading conditions.

Lateral compression measured at 50% tooth height significantly impacted maximum tooth stress in our BTA under loads simulating pulling and shaking. Tooth models with a high degree of lateral compression, like PC1 min, PC1 min & PC2 max, PC1 min & PC2 min, the ocelot and raccoon, recorded higher stresses than those with a low degree, like PC1 max, PC1 max & PC2 min, PC1 max & PC2 max and the Tasmanian devil (figure 4*a*). All regressions between lateral compression and tooth stress showed a significant relationship (*p* < 0.01); however, correlation strength varied among loading conditions (adjusted *R*^2^ values: PULL 50%: 0.520; PULL 90%: 0.497; SHAKE 50%: 0.722; SHAKE 90%: 0.683; electronic supplementary material, table S5). We observed a similar pattern when lateral compression was measured at 90% tooth height (electronic supplementary material, table S5).

##### 3.1.3.2. Robusticity and curvature

**Figure 5.**
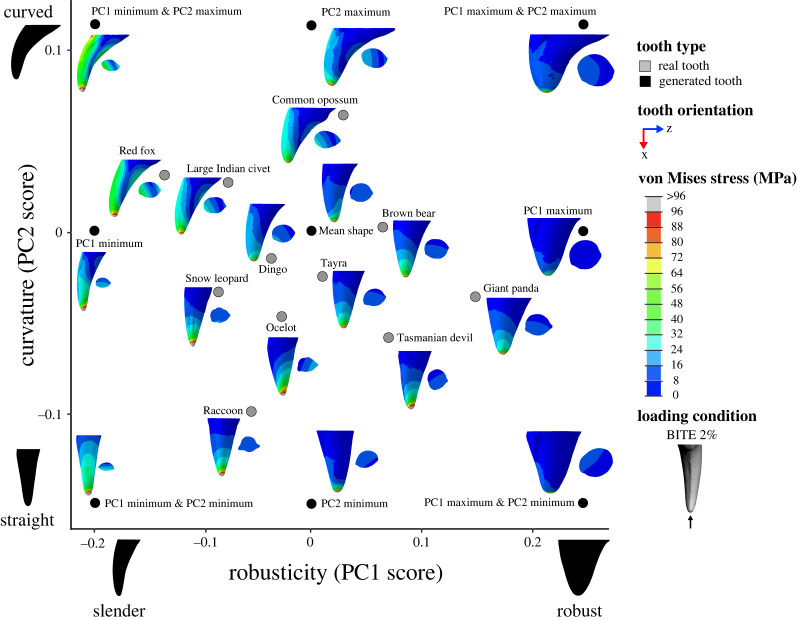
Combined canine tooth three-dimensional geometric morphometric (3DGM) and von Mises stress (MPa) morphospace of real and generated models showing shape variation and stress magnitudes and distributions under a simulated biting load (BITE 2%). The geometric morphometric data and resulting shape space is from Pollock *et al.* [40]. Tooth models in morphospace are scaled to the same tooth height and shown in the lingual view, with cross-section at 50% tooth height. Image to illustrate loading condition in key shown in the posterior view. Contour plots scaled to 96 MPa. Force applied was 300 N.

Robusticity (PC1 score) significantly impacts maximum tooth stress in our BTA under loads simulating pulling and shaking, with slender teeth, like PC1 min, PC1 min & PC2 max, PC1 min & PC2 min, the red fox, and snow leopard, recording high stresses and robust teeth, like PC1 max, PC1 max & PC2 min, PC1 max & PC2 max, the giant panda and brown bear, low stresses (figure 5*b*). We found a significant negative correlation between robusticity and tooth stress across all loading conditions (*p* < 0.01); however, correlation strength varied between loading conditions (adjusted *R*^2^ values: PULL 50%: 0.828; PULL 90%: 0.857; SHAKE 50%: 0.720; SHAKE 90%: 0.751; electronic supplementary material, table S5).

Curvature (PC2 score) did not significantly impact maximum tooth stress for our BTA for any load, with no correlation found between curvature and BTA stress (*p* > 0.47) (figure 5*c*; electronic supplementary material, table S5).

### 3.2. Finite-element analysis (FEA)

#### 3.2.1. Tooth stress among loading conditions

Contour plots reveal that the magnitude and distribution of von Mises stresses varied among loading conditions (figures 5–7; electronic supplementary material, figures S4–S15).

**Figure 6.**
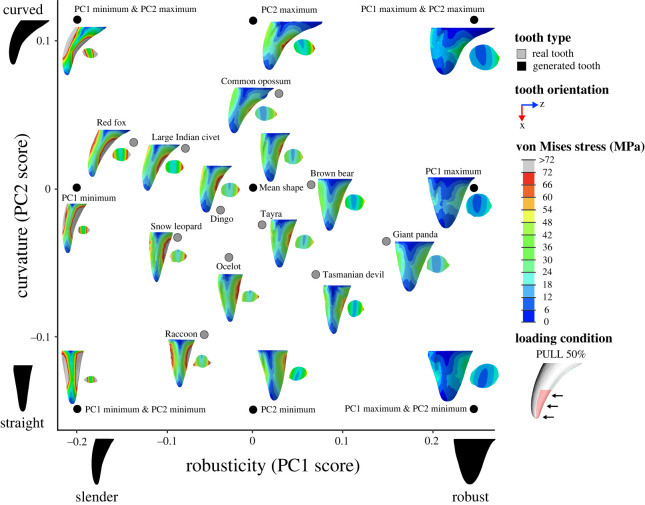
Combined canine tooth three-dimensional geometric morphometric (3DGM) and von Mises stress (MPa) morphospace of real and generated models showing shape variation and stress magnitudes and distributions under a simulated pulling load (PULL 50%). The geometric morphometric data and resulting shape space is from Pollock *et al.* [40]. Tooth models in morphospace are scaled to the same tooth height and shown in the lingual view, with cross-section at 50% tooth height. Image to illustrate loading condition in key shown in the labial view. Contour plots scaled to 72 MPa. Force applied was 300 N.

**Figure 7.**
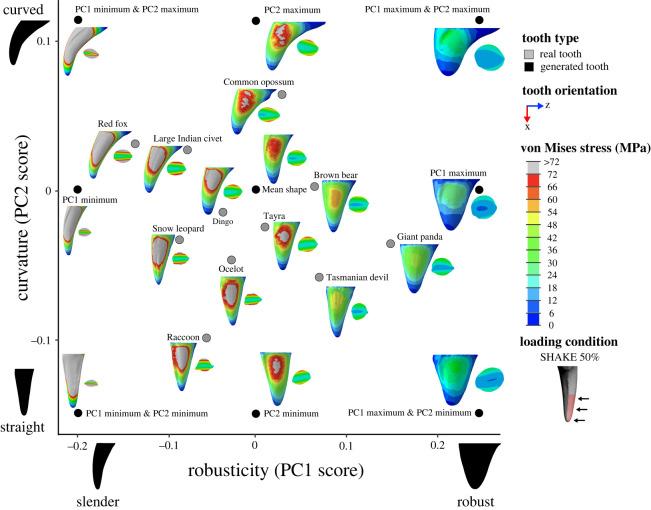
Combined canine tooth three-dimensional geometric morphometric (3DGM) and von Mises stress (MPa) morphospace of real and generated models showing shape variation and stress magnitudes and distributions under a simulated shaking load (SHAKE 50%). The geometric morphometric data and resulting shape space is from Pollock *et al.* [40]. Tooth models in morphospace are scaled to the same tooth height and shown in the lingual view, with cross-section at 50% tooth height. Image to illustrate loading condition in key shown in the posterior view. Contour plots scaled to 72 MPa. Force applied was 300 N.

For load BITE 2%, von Mises stress magnitudes were concentrated at the tip of the tooth with the highest magnitudes (≥96 MPa) at the tip and the lowest (approx. 0 MPa) at the base (figure 5; electronic supplementary material, figures S4 and S5). In some tooth models, like PC1 max, the raccoon, Tasmanian devil, giant panda and snow leopard, von Mises stresses were distributed relatively evenly around the central axis of the tooth. However, in other tooth models, like PC1 min & PC2 max, PC2 max, the red fox, large Indian civet, common opossum and dingo, the anterior surface of the tooth experienced higher von Mises stress magnitudes (approx. 24 to 48 MPa) than other surfaces (approx. 0 to 24 MPa). We also observed von Mises stress hot-spots (approx. 16 to 48 MPa) on the upper posterior surface of PC1 min & PC2 max, PC2 max, the red fox, large Indian civet and common opossum. When a larger surface area of tooth was loaded, as in the BITE 50% condition, the distribution of von Mises stresses differed, whereby the highest magnitudes (≥24 MPa) were not observed at the tip of the tooth models, but at their midsections (electronic supplementary material, figures S6 and S7). The tip and base of the tooth models experienced some of the lowest von Mises stress magnitudes (approx. 0 MPa). However, the same tooth models (PC1 min & PC2 max, PC2 max, the red fox, large Indian civet, common opossum) still experienced higher von Mises stress magnitudes (approx. 10 to ≥24 MPa) at their anterior surfaces and on their posterior surface (≥24 MPa), similar to load BITE 2%.

**Figure 8.**
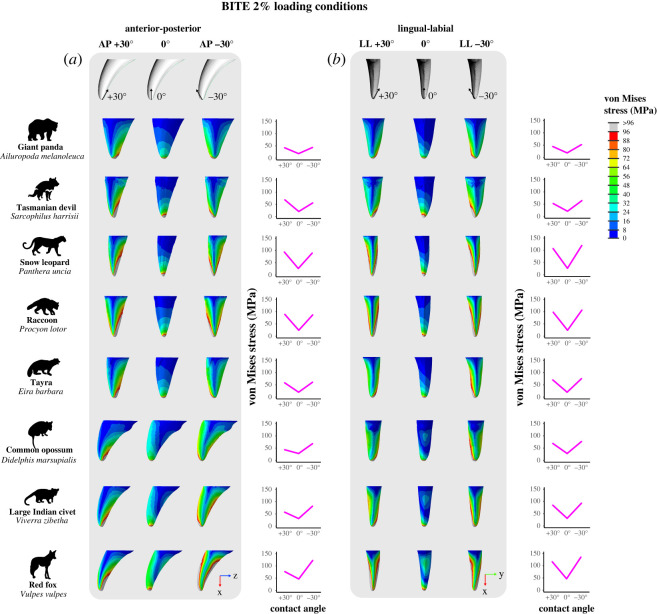
Contour plots showing magnitude and distribution of von Mises (MPa) stresses and line graphs showing maximum stress values generated from finite-element analysis (FEA) in a selection of real tooth models under loading conditions simulating initial biting where the contact angle between the tip of the tooth and the prey vary from perpendicular to the prey (BITE 2% 0°) to off-angle in either the anterior–posterior (BITE 2% AP +30°, BITE 2% AP −30°) or lingual labial directions (BITE 2% LL +30°, BITE 2% LL −30°). (*a*) Varying the contact angle between tooth and prey in the anterior–posterior direction with tooth images scaled to the same tooth height and shown in the lingual view. (*b*) Varying the contact angle between tooth and prey in the lingual–labial direction with tooth images scaled to the same tooth height and shown in the posterior view. FEA maximum stress value for each tooth model is the 90^th^ percentile of von Mises stress values for all surface nodes. Force applied was 300 N for BTA and FEA under all loading conditions.

Varying the contact angle between the tooth and prey during initial biting (BITE 2%) impacted the magnitude and distribution of von Mises stresses (figure 8*a* and *b*). When the tip of the tooth was perpendicular to the food (0°, as in BITE 2%) the highest stress magnitudes were concentrated at the tip, decreasing as they approached the base and mostly distributed evenly around the central axis of the tooth. However, this changed for all off-angle biting simulations. When varying the contact angle in the anterior–posterior direction this changed, so in the BITE 2% AP +30° loading condition the highest magnitudes (≥96 MPa) extended up from the tip of the tooth along the posterior edge, while the lowest magnitudes (approx. 0 MPa) were observed on the lingual and labial surfaces starting close to the tip and extending to the base. Von Mises stresses are not distributed evenly around the central axis of the tooth and are concentrated on the anterior and posterior surfaces (figure 8*a*). In some tooth models, like PC1 min & PC2 min, PC2 min, the raccoon, and snow leopard, the stress magnitudes on the anterior and posterior surfaces are roughly equivalent (approx. 32 to ≥96 MPa). However, in other tooth models, like PC1 min & PC2 max, PC2 max, the common opossum, large Indian civet, and red fox, the anterior surface shows lower stress magnitudes (approx. 8 to ≥48 MPa) than the posterior (approx. 32 to ≥96 MPa). In the BITE 2% AP −30° loading condition we observed a similar pattern, with the lowest von Mises stress magnitudes (approx. 0 MPa) observed on the lingual and labial surfaces. The highest magnitudes (≥96 MPa) are also found at the tip of the tooth; in most models, however, they extend along the anterior edge (figure 8*b*). In some tooth models, like PC1 min & PC2 min, PC1 min & PC2 max, PC2 min, the snow leopard, raccoon, large Indian civet, and red fox, high stress magnitudes (approx. 80 to ≥96 MPa) are also found on the posterior edge. Von Mises stresses are concentrated on the anterior and posterior surfaces, and for most tooth models the magnitudes on these surfaces are roughly equivalent (approx. 32 to ≥96 MPa). The von Mises stress magnitudes experienced between the BITE 2% AP +30° and BITE 2% AP −30° loading conditions were roughly equivalent for most tooth models; however, for some (PC1 min & PC2 max, PC2 max, the common opossum, large Indian civet and red fox) BITE 2% AP −30° produced higher stress magnitudes (figure 8*a*).

In the BITE 2% LL +30° loading condition, the highest magnitudes (≥96 MPa) extended up from the tip of the tooth along the labial surface. The lowest magnitudes (approx. 0 to 32 MPa) were observed on the anterior and posterior edges starting close to the tip and extending to the base. Von Mises stresses are concentrated on the lingual and labial surfaces, and their magnitudes roughly equivalent (approx. 32 to ≥96 MPa) (figure 8*b*). We observed the same pattern in the BITE 2% LL −30° condition; however, highest magnitudes (≥96 MPa) extended up from the tip of the tooth along the lingual surface (figure 8*b*). The von Mises stress magnitudes experienced between the BITE 2% LL +30° and BITE 2% LL −30° loading conditions were roughly equivalent for all tooth models (figure 8*b*).

For load PULL 50%, the highest von Mises stress magnitudes (≥72 MPa) were observed at the posterior and anterior surfaces of the tooth models, while the lowest (approx. 0 to 6 MPa) were observed at the tip, base, and lingual and labial surfaces (figure 6; electronic supplementary material, figures S8 and S9). This pattern of von Mises stress distribution remained relatively consistent among all tooth models and also when a larger surface area of tooth was loaded (PULL 90%) (electronic supplementary material, figures S10 and S11).

By contrast, under SHAKE 50% the highest von Mises stress magnitudes (≥72 MPa) were observed at the labial and lingual surfaces of the tooth models, while the lowest (approx. 0 to 12 MPa) were observed at the tip, base and anterior and posterior surfaces (figure 7; electronic supplementary material, figures S12 and S13). This pattern of von Mises stress distribution also remained relatively consistent among all tooth models and when a larger surface area of tooth was loaded (SHAKE 90%) (electronic supplementary material, figures S14 and S15).

#### 3.2.2. Tooth stress among models

Comparison of contour plots across tooth models shows that the von Mises stress magnitudes vary among models (figures 5–7: electronic supplementary material, figures S4–S15). By considering these in conjunction with the maximum von Mises stress values we can determine which of our tooth models experienced high and low von Mises stresses.

Of the real tooth models tested in our FEA, the red fox appears to experience the highest magnitude of von Mises stresses under all loading conditions (approx. 24 to ≥ 96 MPa) (figures 5–7; electronic supplementary material, figures S4–S15); this was supported by the maximum von Mises stress values (figure 3*b*). Other tooth models that appear to experience high von Mises stress magnitudes were the large Indian civet, snow leopard and raccoon (figures 5–7; electronic supplementary material, figures S4–S15). The lowest von Mises stress magnitudes were seen in the giant panda for all loading conditions (approx. 8 to ≥96 MPa, although ≥96 MPa was only observed at the tooth tip under load BITE 2%; for all other loading conditions the von Mises stress magnitudes ranged between approximately 8 and 48 MPa) (figures 5–7; electronic supplementary material, figures S4–S15); this was also supported by the maximum von Mises stress values (figure 3*b*). Other tooth models that experienced low stress magnitudes were the brown bear and Tasmanian devil (figures 5–7; electronic supplementary material, figures S4–S15).

The generated tooth model that experienced the highest von Mises stress magnitude varied among loading conditions. For example, for all PULL and SHAKE loads, PC1 min, PC1 min & PC2 min and PC1 min & PC2 max appear to experience roughly the same high von Mises stress magnitudes (figures 5–7; electronic supplementary material, figures S4–S15); this is also reflected in the maximum von Mises stress values, which are similar among the three models (figure 3*b*). By contrast, under BITE loads PC1 min & PC2 max appeared to have the highest von Mises stress magnitude of all generated tooth models, apart from the BITE 2% LL loads, where PC1 min & PC2 min was higher (figure 5; electronic supplementary material, figures S4–S15); this was supported by the maximum von Mises stress value (figure 3*b*). It was hard to determine which model experienced the lowest von Mises stress magnitude, as PC1 max, PC1 max & PC2 max and PC1 max & PC2 min showed similar magnitudes under all loading conditions as well as similar maximum von Mises stress values (figure 3*b*); this was consistent among all loading conditions.

#### 3.2.3. Tooth stress and canine shape

##### 3.2.3.1. Lateral compression

Inspection of contour plots shows that von Mises stress magnitude appears to be correlated with the degree of lateral compression, whereby tooth models with a high degree of lateral compression, like PC1 min & PC2 min, PC1 min & PC2 max, PC1 min, the raccoon and ocelot, all experience higher stress magnitudes (approx. 24 to ≥96 MPa) than models with a low degree of lateral compression, like PC1 max, PC1 max & PC2 min, PC1 max & PC2 max and the Tasmanian devil (approx. 0 to 60 MPa) (figures 6–8; electronic supplementary material, figures S4–S15). This is consistent among all loading conditions and is supported by regressions between lateral compression measured at 50% of tooth height and maximum von Mises stress, which, except for load BITE 50% (*p* = 0.06), were all significant (*p* < 0.03); however, correlation strength varied considerably (adjusted *R*^2^: BITE 2%: 0.280; BITE 2% AP +30°: 0.590; BITE 2% AP −30°: 0.475; BITE 2% LL +30°: 0.763; BITE 2% LL −30°: 0.729; PULL 50%: 0.587; PULL 90%: 0.625; SHAKE 50%: 0.687, SHAKE 90%: 0.676; figure 4*d*; electronic supplementary material, table S7). We observed a similar pattern when lateral compression was measured at 90% tooth height (electronic supplementary material, table S7).

##### 3.2.3.2. Robusticity

We can also see a pattern for robusticity. Slender canines, like PC1 min & PC2 min, PC1 min & PC2 max, PC1 min, the red fox, large Indian civet and snow leopard, show higher von Mises stress magnitudes. By contrast, robust models, like PC1 max, PC1 max & PC2 min, PC1 max & PC2 max, the giant panda, brown bear and Tasmanian devil, show lower von Mises stress magnitudes (figures 5–7; electronic supplementary material, figures S4–S15). This pattern is also supported by the regressions between robusticity and maximum von Mises stress, which were significant under all loading conditions (*p* < 0.01); however, correlation strength varied (adjusted R^2^: BITE 2%: 0.655; BITE 2% AP +30°: 0.762; BITE 2% AP −30°: 0.813; BITE 2% LL +30°: 0.717; BITE 2% LL −30°: 0.730; BITE 50%: 0.500; PULL 50%: 0.837; PULL 90%: 0.819; SHAKE 50%: 0.720, SHAKE 90%: 0.742; figure 4*e*; electronic supplementary material, table S7).

##### 3.2.3.3. Curvature

We do not observe a pattern between curvature and von Mises stress magnitude under PULL 50%, PULL 90%, SHAKE 50% or SHAKE 90% loading conditions (figures 6 and 7; electronic supplementary material, figures S8–S15). This is confirmed by the regressions between curvature and maximum von Mises stress, which were not significant for any PULL or SHAKE loading conditions (*p* > 0.50) (figure 4*f*; electronic supplementary material, table S7). By contrast, for BITE 2% and BITE 50% loading conditions we can see a pattern in stress magnitude, where curved teeth, like the PC1 min & PC2 max, PC2 max, the common opossum and red fox, appear to experience higher von Mises stress magnitudes (≥20 MPa) (especially on their anterior surfaces) than straight models, like PC2 min, the raccoon and Tasmanian devil (figure 5; electronic supplementary material, figures S4–S7). However, this pattern was not supported by the regressions between curvature and maximum von Mises stress, which were not significant for any BITE loading conditions (*p* > 0.73, figure 4*f*; electronic supplementary material, table S7). In addition, when we compare all off-angle biting loading conditions (BITE 2% AP +30°, BITE 2% AP −30°, BITE 2% LL +30° and BITE 2% LL −30°) there is a pattern in stress magnitude, where curved teeth under the BITE 2% AP +30° load experience comparatively lower magnitudes than all others. Straight teeth, on the other hand, experience approximately the same stress magnitudes under all off-angle biting loads (electronic supplementary material, figure S16).

### 3.3. Deformation

**Figure 9.**
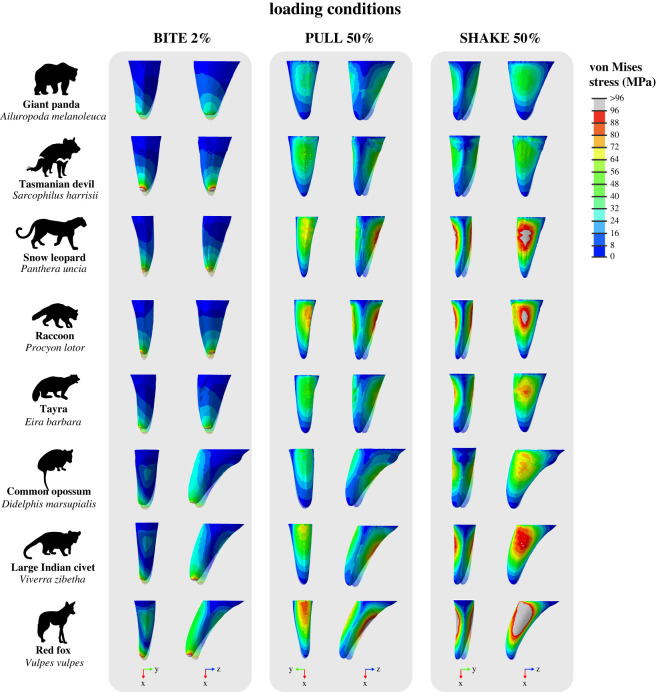
Patterns of deformation and von Mises (MPa) stress distribution in a selection of real tooth models under loading conditions BITE 2%, PULL 50%, and SHAKE 50% from Finite-Element Analysis (FEA). Top to bottom showing the giant panda (*Ailuropoda melanoleuca*), raccoon (*Procyon lotor*), tayra (*Eira barbara*), common opossum (*Didelphis marsupialis*) and red fox (*Vulpes vulpes*). All tooth models are scaled to the same tooth height. For loading conditions BITE 2% and SHAKE 50%, tooth is in posterior (left) and lingual views (right). For loading condition PULL 50%, tooth is in anterior (left) and lingual views (right). Von Mises contour plot is scaled to 96 MPa. Deformation factors shown here were auto-computed for each unique combination of tooth model and loading condition in Abaqus to better illustrate the structural response to loads and for ease of comparison. Force applied was 300 N.

There are some general patterns that emerged when mapping the deformation of a selection of tooth models (figure 9). When under PULL 50% and SHAKE 50% loading conditions, we see the same patterns of deformation among all tooth models, where deformation occurred primarily in the direction force is applied. Under PULL 50% deformation occurred in the anterior direction and under SHAKE 50% deformation occurred in the lingual direction (figure 9).

Like PULL 50% and SHAKE 50% loading conditions, when under BITE 2% loads, all tooth models deformed in the direction force is applied. However, depending on the degree of curvature, some tooth models also deformed in other directions. Straight (giant panda and raccoon), intermediate (tayra) and curved teeth (common opossum and red fox) all deformed in the dorsal direction, and apart from the raccoon, exhibit a small amount of deformation in the lingual direction (figure 9). In addition to this, curved teeth also deformed in the anterior direction.

Tooth robusticity did not appear to impact the direction of deformation under any loading condition (figure 9). However, it did impact the degree of deformation, whereby robust teeth (giant panda) deformed to a lesser degree than slender teeth (red fox) for a given fixed deformation factor (electronic supplementary material, figure S17).

### 3.4. Canine shape, stress and feeding ecology in a comparative context

**Figure 10.**
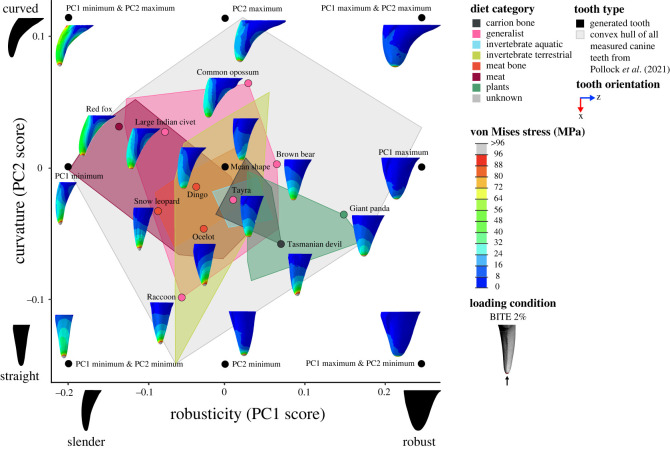
Combined canine tooth three-dimensional geometric morphometric (3DGM) and von Mises stress (MPa) morphospace of real and generated models showing shape variation and stress magnitudes and distributions under a simulated biting load (BITE 2%). The geometric morphometric data and resulting shape space, overlain diet categories, and convex hulls are from Pollock *et al.* [40]. Tooth models in morphospace are scaled to the same tooth height and shown in the lingual view. Grey convex hull generated from all canine teeth both upper and lower, diet convex hulls generated from only upper canine teeth. Contour plots scaled to 96 MPa. Force applied was 300 N.

Inspection of the combined 3DGM morphospaces with overlaid diet and killing technique categories reveal links between canine shape, tooth stress and feeding ecology. The BITE 2% loading condition with overlaid diet categories shows that slender teeth, like the red fox, snow leopard and large Indian civet, experience relatively high-stress magnitudes and are associated with meat, meat bone and generalist diets. By contrast, robust teeth like giant panda, Tasmanian devil and brown bear experience relatively low-stress magnitudes and are associated with plant, carrion bone and generalist diets. Additionally, both curved and straight teeth (common opossum and raccoon, respectively) are associated with generalist diets (figure 10).

**Figure 11.**
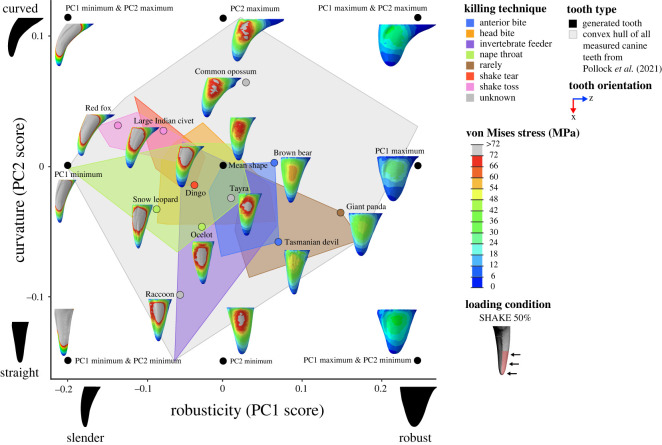
Combined canine tooth three-dimensional geometric morphometric (3DGM) and von Mises stress (MPa) morphospace of real and generated models showing shape variation and stress magnitudes and distributions under a simulated shaking load (SHAKE 50%). The geometric morphometric data and resulting shape space, overlain diet categories, and convex hulls are from Pollock *et al.* [40]. Tooth models in morphospace are scaled to the same tooth height and shown in the lingual view. Grey convex hull generated from all canine teeth both upper and lower, killing convex hulls generated from only upper canine teeth. Contour plots scaled to 72 MPa. Force applied was 300 N.

Both PULL 50% and SHAKE 50% loading conditions with overlaid killing technique categories show that slender teeth, like those of the red fox, snow leopard and large Indian civet, experience relatively high-stress magnitudes and are associated with nape throat and shake toss killing techniques. Robust teeth, like the giant panda, Tasmanian devil and brown bear, experience relatively low-stress magnitudes and are associated with carnivores that rarely kill prey and anterior bite-killing techniques. In addition, curved and slender teeth, like those of the red fox and large Indian civet, are associated with shaking killing techniques, while straight and slender teeth, like those of the snow leopard and ocelot, are associated with the nape throat killing technique (figure 11; electronic supplementary material, figure S18).

### 3.5. Comparison of BTA and FEA models

All regressions between calculated BTA maximum stress and FEA von Mises stress were significant (*p* < 0.01) and showed a high degree of correlation (adjusted *R*^2^: SHAKE 50%: 0.982; SHAKE 90%: 0.987, PULL 50%; 0.961; PULL 90%: 0.927; electronic supplementary material, figure S19).

## 4. Discussion

The variation in canine shape present among mammalian carnivores impacts stresses during simulated killing and feeding. In this study, we are able to quantify how key aspects of canine tooth shape—robusticity, curvature and lateral compression—affect a tooth's ability to withstand stresses produced under loading conditions simulating biting, pulling and shaking. Some aspects of shape, like robusticity and lateral compression, impacted von Mises stress magnitude and distribution and were significantly correlated with BTA and FEA measures of maximum tooth stress. As robusticity increased, von Mises stress magnitudes and maximum stress values decreased for all loading conditions, a pattern that appears to be related to the material properties of the prey handled. The degree of tooth lateral compression, on the other hand, determined relative ability of canines to resist forces in multiple directions, whereby canines with more circular cross-sections experienced roughly equal maximum stresses when pulling and shaking, while more laterally compressed canines experienced higher maximum stresses when shaking. Other aspects of shape, like curvature, surprisingly had no effect on maximum tooth stress; however, they did impact von Mises stress distribution and tooth deformation when under loading conditions simulating biting. We were also able to observe general patterns among loading conditions; for example, shaking produced the highest maximum stresses for almost all teeth, followed by pulling, and then biting. In addition, for all tooth models, off-angle biting loading conditions produced higher maximum stresses than those where the tip of the tooth was perpendicular to the prey.

### 4.1. The relationship between canine shape, tooth stress and feeding ecology

#### 4.1.1. Lateral compression

Our results show that the degree of tooth lateral compression significantly impacts tooth stress under loading conditions simulating pulling and shaking. In both our BTA and FEA simulations we found that tooth models with a high degree of lateral compression (more elliptical in cross-section) recorded higher maximum stresses than those with a low degree of lateral compression (more circular in cross-section). However, the advantage of the lateral compression metric lies in its ability to determine the relative stresses a canine tooth experiences when pulling and shaking. In line with previous studies, we found canine teeth that are more circular in cross-section experience relatively equal stresses under loads simulating pulling and shaking than those that are more elliptical [4]. By also applying FEA we were able to show that this pattern holds true when modelling canine teeth in three dimensions, including curved teeth (figure 4).

The Tasmanian devil illustrates this pattern nicely, showing the lowest degree of lateral compression of all the teeth measured (almost a 1:1 ratio of anterior–posterior width / lingual–labial width) and experience almost equal maximum stresses (for both BTA and FEA) when pulling and shaking (electronic supplementary material, figure S3). In fact, the devil is the only example of a canine tooth that is wider in the lingual–labial axis than the anterior–posterior axis. All canine teeth measured by Van Valkenburgh & Ruff [4], Christiansen [6,24] and Christiansen & Adolfssen [26] showed some degree of lateral compression. Devils are scavengers that regularly consume hard bone and employ a range of prey-processing techniques that expose their teeth to unpredictable loads [40,58–60]. In addition to having the highest predicted bite force of any extant mammal when scaled for body size [61], Van Valkenburgh & Ruff [4] also proposed that the relatively circular cross-sections of hyaenas were an adaptation to scavenging, and our observations of devil canines support this.

#### 4.1.2. Robusticity

Canine robusticity significantly impacts both BTA and FEA maximum tooth stresses under all loading conditions, with slender teeth recording high stresses and robust teeth low stresses. We found that, regardless of the loading condition (BITE, PULL or SHAKE), robust canine teeth experienced lower maximum stresses than slender canine teeth. This implies that, during feeding, for a given applied force, robust canine teeth have a lower risk of breakage relative to slender teeth. Pollock, Hocking [5] found a correlation between robust canines and biting into 'hard' prey. Our combined 3DGM, stress and diet morphospace (figure 10) suggests that these correlations between prey material properties and canine robusticity are related to a tooth's ability to tolerate the stresses associated with biting into different prey. It appears that robust teeth can better tolerate the stresses that result from the high unpredictable forces associated with regularly encountering bone or varied 'hard' foods [4]. By contrast, although slender canines experience higher stresses given the same load, they can be tolerated as they predominantly bite into muscle and flesh with a relatively low chance of encountering bone. In an ecological context, habitual consumers of bone, like Tasmanian devils, have relatively robust canines that experience comparatively low von Mises stress magnitudes [62,63], while the slender canines of the red fox primarily encounter muscle and flesh and experience relatively high von Mises stress magnitudes [64]. However, we also found the ecology that underlies this pattern can be variable: more generalist feeders like the brown bear and the tayra experience von Mises stress magnitudes similar to the Tasmanian devil when biting (with similar robusticity) (figure 10); however, they are not specialized carcass scavengers [65,66]. Another example is the snow leopard and raccoon, which have similar stress magnitudes and similar robusticity to one another, yet the snow leopard is a targeted nape throat killer that primarily consumes muscle with some large bones and the raccoon is a generalist that can eat anything from plant material to insects and small mammals [20,67,68].

In multi-part structures, like the vertebrate jaw, redundant linkages between form and function have been attributed to the fact that complex systems are subject to environmental, developmental and functional trade-offs that may constrain optimization of one or a few mechanical traits [69–71]. When placed in the context of the mammalian jaw, canine teeth demonstrate an additional level of many-to-one mapping of form to function [70]. Our results indicate that even in relatively simple structures, like canines, the simultaneous biomechanical demands of prey capture and consumption can result in multifaceted form-function relationships. When considering these biomechanical demands, the ability of a canine tooth to resist stresses associated with handling prey with specific material properties is only one side of the story. The ability to puncture prey with specific material properties is equally important. For example, although robust canines are less likely to break when encountering bone or other hard foods when biting into deformable materials like muscle, slender teeth can puncture more effectively as, for a given depth, there is a smaller volume of tooth in the prey, decreasing the force required to puncture [23,46]. When considering the optimal shape of a canine tooth there is a trade-off at play between tooth penetration and breakage risk, and these factors are dependent on the material properties of the prey being handled [23,46]. Our study only presents one side of this trade-off: the relative stresses a tooth experiences under different loading conditions. In the future, we will conduct a series of physical performance tests with the same tooth models to investigate puncture and further elucidate the relationship between tooth shape, stress, puncture and the material properties of prey.

An additional point to explore in future studies is the relationship between the bite force and tooth stress for specific species. Our simulations applied the same load (300 N) across scaled tooth models enabling us to look at relationships between shape and stress in a comparative form–function context. This approach does not take into account the bite force a species is able to exert on a tooth of a given size and shape. For example, a giant panda has robust canines and a higher bite force than a red fox [53]. It may be that when unscaled teeth are loaded with the relevant bite force they experience similar stresses; however, this remains to be tested.

#### 4.1.3. Curvature

Curvature did not significantly impact maximum tooth stress in any of the loading conditions tested for both BTA and FEA. This is surprising, given that past research on canine tooth shape found a correlation between the degree of curvature and shaking killing behaviours [5]. We found no relationship between curvature and BTA or FEA maximum stress under loads simulating shaking. In fact, the combined 3DGM, shaking stress and killing technique morphospace (figure 11) shows that species which are known to shake their prey, like the red fox and large Indian civet, have curved and slender canines that experience some of the highest maximum stresses when shaking [20,22,72]. This may seem contradictory; however, it may be related to the size of the prey being shaken. Shaking behaviours are generally only performed on smaller prey, and so the force acting on the teeth is expected to be relatively low [22]. Hence, tolerating stresses associated with shaking larger prey may not be essential. A further point to consider is the biting depth at which shaking behaviours may be performed. If they are predominantly performed with the whole tooth embedded in the prey, additional teeth like the incisors will also be engaged and would likely impact stresses.

Although we did not find a direct correlation between curvature and maximum tooth stress, our FEA contour plots reveal that curvature impacts the magnitude and distribution of von Mises stresses under loads simulating initial biting (BITE 2%). For biting loads where the tip of the tooth was perpendicular to the prey (0°), stress magnitudes increased with curvature, primarily distributed on the anterior surface of the tooth (figure 5). Curvature also impacted a tooth's ability to tolerate stresses experienced during ‘off-angle’ biting, where the tip of the tooth is not perpendicular to the food (AP +30°, AP −30°, LL +30°, and LL −30°) (figure 9). It appears that canine curvature is especially relevant when biting, such that straight and curved teeth may be suited to different feeding scenarios. Straight canines, like the raccoon and Tasmanian devil, appear to be ‘all-rounders’, experiencing lower stress magnitudes that are more centrally distributed, when biting perpendicular to the prey, and approximately equal magnitudes among off-angle loading conditions. By contrast, curved canines, like the red fox and common opossum, appear more specialized: when biting perpendicular to the prey they experience higher stress magnitudes that are anteriorly distributed and among off-angle conditions comparatively lower magnitudes under the AP +30° load. Varying the contact angle in the anterior–posterior direction is likely related to the size of the prey being bitten into or the approach angle of the predator to the prey. This has been investigated in snakes [12,73,74]; however, it requires further study in mammalian carnivores.

By modelling how a selection of canines deform under loads simulating initial biting (BITE 2%; figure 9), we found that curvature also impacted the axes in which bending occurred, whereby straight and curved teeth differ primarily in their deformation along the anterior–posterior *z*-axis. Straight canines did not deform along the anterior–posterior axis; their primary axis of deformation was in the dorsal direction. Curved canines, however, did deform along the anterior direction as well as in the dorsal direction, *x*-axis. This difference in deformation between straight and curved teeth is likely to do with how closely the central axis of the tooth matches the direction of force when biting [12]. In straight canines, these are essentially parallel, producing an even distribution of stress around the central axis of the tooth, where the tip region of the tooth is under compression, deforming a straight tooth in the direction of force (figure 9). However, in curved canines, the direction of force does not match the central axis of the tooth as closely, producing an uneven distribution of stress (figure 9). Here, both the tip and anterior regions of the tooth are likely under compression and the posterior region under tension causing a curved tooth to deform in multiple axes.

### 4.2. Canine tooth stress among loading conditions

A canine tooth's primary function is to bite into prey, loading the dorsal–ventral axis of the tooth [3,20]. Functions that load the canine tooth along the anterior–posterior axis are also relatively common; for example, tearing off pieces of prey is common to almost all mammalian carnivores [3,4,20–22,72,75–79]. Functions that load canine teeth along the lingual–labial axis, like shaking or subduing struggling prey, are less common [4,20,80]. We observed a general pattern, where loading conditions simulating biting produced the lowest maximum FEA von Mises stresses in all tooth models, followed by pulling loads, and shaking loads (figure 3*b*). This indicates that, for a given force (300 N), canine teeth are least likely to break under loading conditions simulating biting, where the force is directed along the dorsal–ventral axis, and more so under pulling and shaking loads, where the force is directed along the anterior–posterior or lingual–labial axes, respectively. We propose this is related to the relative frequency these axes are loaded during killing and feeding, whereby the direction in which canine teeth are strongest (experience the lowest stresses) is the one most regularly loaded. This relationship has been suggested previously, but primarily in relation to the degree of lateral compression and tolerance of lateral loads via BTA [4,27,28,81].

We also observed a general pattern for all tooth models among biting loads, where conditions simulating off-angle biting produced higher maximum stresses than those where the tip of the tooth was perpendicular to the prey. This suggests that the contact angle between a canine tooth and prey is important for reducing the stress experienced by a canine tooth, whereby the closer the tip of the tooth is to being perpendicular to the prey, the lower the chance of breakage. However, in practice, very little is known about how much this can vary during killing and feeding in carnivores. This should be investigated in future research.

### 4.3. Implications for tooth breakage

Our results show how aspects of canine shape and simulated feeding behaviours can impact the stresses experienced by a tooth, enabling us to highlight relationships between tooth morphology and feeding ecology that help predators target their preferred prey, while minimizing the likelihood of breakage. However, in practice, these relationships between form and function are not absolute. Tooth breakage in carnivores is common, especially for canines, which are the most frequently broken tooth type [9,82]. From breakage frequencies in wild individuals, it is clear that throughout an animal's life, canine teeth are often exceeding their mechanical capacity. For example, the slender curved canines of red foxes can have relatively high incidences of breakage, so too can the comparatively robust canines of the spotted hyaena (*Crocuta crocuta*) [9,82,83]. From our simulations, we can identify potential scenarios where canine forms are most likely to break (experience the highest von Mises stresses), such as off-angle biting especially in the lingual–labial direction and under lateral loads, particularly shaking. This is also the case in slender canines biting into 'hard' prey or curved slender canines during off-angle biting, specifically in the posterior direction. For some of these scenarios, like shaking, it is important to note that the loads can be intrinsic (generated by the predator) or extrinsic (generated by the prey). Biting into and shaking prey or handling struggling prey may produce lateral loads and high stresses that increase the likelihood of breakage. Our simulations can also help to pinpoint the areas on a canine tooth most likely to break (regions with the highest von Mises stresses). For example, from the distribution of stresses in canines under biting loads we might expect curved teeth to break at their tip, along the anterior edge, or for canines under lateral loads to exhibit transverse fracture [39,84]. However, this requires further study and validation through closer inspection of specimens with broken teeth to identify the type and location of breakage, in addition to *in vivo* breakage testing of real teeth under controlled conditions. This type of work has been undertaken on domestic dog (*Canis familiarus*) canines, with patterns observed that support some of the above predictions [28,81]. For example, a trend toward lower force to fracture (N) when teeth are loaded in the lingual–labial direction [81], in addition to transverse fracture being one of the most common fracture types under lingual–labial loading [81]. Future research should expand on these studies by including a broader range of canine forms.

### 4.4. Modelling canine tooth stress with beam theory and finite-element analysis

The maximum stresses calculated using BTA and FEA under loading conditions simulating pulling and shaking are highly correlated and, overall, we see the same general patterns across all tooth models using both these methodologies. This is somewhat surprising given the simplicity of BTA and relative complexity of FEA. However, the constraints and loads we applied in our FEA simulations closely matched those of the assumptions made in BTA [8,23,85]. We essentially modelled canines as a cantilever beam in our FEA simulations, with their constrained dorsal surface and application of lateral loads. It is useful to highlight that we observed similar results regardless of modelling the canine tooth as a simple solid cylinder with BTA or as a complex three-dimensional shape with FEA. If researchers are presented with time or computing constraints, BTA is still a useful and informative approach for modelling aspects of canine tooth biomechanics.

The utility of FEA is that it offers a more realistic approach to investigate the nuanced aspects of the relationship between canine tooth shape and stress [8]. For example, initially with BTA we found no correlation between curvature and maximum tooth stress. However, by applying FEA and modelling von Mises stress distributions and tooth deformation we were able to observe how curvature impacts the areas of a tooth that experience high magnitudes of stress, in addition to the directions in which the tooth bends under different loading conditions. These are insights that would not be possible with BTA alone. Another example of the utility of FEA is its ability to model the impact of other aspects of tooth shape like sharpness, as demonstrated on shark teeth by Whitenack, Simkins Jr [38]. Although investigating sharpness fell outside the scope of this study, our results indicate that it impacts tooth stress and that this is best captured by FEA. If we consider the raccoon canine, BTA is unable to model the sharp anterior and posterior edges it possesses and, as a result, underestimates the maximum stresses it experiences under loads simulating pulling (electronic supplementary material, figure S21). Canine tooth sharpness is important for initial fracture and crack propagation and is correlated with specific diets and feeding ecologies [5,7,46,86–90]. The impact it has on canine tooth stress should be investigated in future studies.

There are additional factors not modelled in this study that should also be considered, like the internal structure of the tooth. As our study focused on the general aspects of the relationship between overall tooth shape and stress, we gave the teeth uniform material properties and did not model the enamel, dentine, pulp cavity layers or root. Exactly how these would impact the observed relationships between canine shape and stress is not known in detail, although enamel has been identified as an important factor for fracture [39]. In addition, the mechanical properties of these internal structures, like dentine has been found to vary with tooth height [91]. Future studies should employ micro-CT to create canine tooth models with accurate differences in the thickness and distribution of enamel and dentine or the size of the pulp cavity, which may help to reveal structural mechanisms among species that help counter stresses, similar to previous studies on fish and reptile taxa, and humans [38,92,93]. Moreover, the path of a canine tooth during a bite is not unidirectional, as we modelled in our static simulations. As the jaws close, they generally follow an arc. This would influence the force and direction of contact between predator and prey and likely impact how they distribute stresses and penetrate prey. In the future, dynamic FEA simulations should be undertaken to model this, whereby the direction of force would change iteratively to simulate the more arc-like movement of the canine as it bites into prey. Moreover, as canines form an integrated part of the mammalian carnivore craniodental system, it is important to consider not just the tooth, but the cranium as well. Future research should focus on building more biologically relevant simulations to investigate how canine teeth function in a broader context.

## 5. Conclusion

In this study, we were able to quantify how canine tooth shape impacts the stresses produced under conditions simulating biting into, pulling apart, or shaking prey and how this relates to carnivore feeding ecology. Our findings help to elucidate the relationships between aspects of canine tooth shape and specific diets or killing techniques from previous research by revealing how differently shaped teeth handle the stresses associated with distinct feeding behaviours [4–6]. We found that some aspects of canine shape, like robusticity and lateral compression, are correlated with maximum tooth stress and this likely reflects the material properties of prey handled or the probability of encountering lateral forces. Interestingly, for all loading conditions, we found that regardless of having a straight or curved tooth, the maximum stresses experienced did not vary significantly. However, curvature did impact stress distribution and tooth deformation under loading conditions simulating biting. We also found general patterns among loading conditions that suggest the overall shape of a canine tooth reflects the relative forces encountered during killing and feeding [4]. In addition, our simulations allowed us to identify feeding scenarios where canine forms are likely to break, as well as pinpoint the areas on a tooth where breakage may occur. This study also demonstrates the utility of FEA for making more nuanced assessments of canine tooth biomechanics and highlight it as an important avenue for future research building more biologically accurate models and simulations [8].

Our findings reveal how the canines of mammalian carnivores have been adapted to tolerate the stresses associated with killing and feeding and enable us to better understand the form–function relationships that drive the diversity of mammalian carnivore feeding ecologies. Moreover, these relationships between aspects of shape in pointed structures and stress may be broadly applicable to other biological structures used for puncture, like claws, simple teeth, snake and spider fangs, or even spines.

## Data Availability

The datasets supporting this article have been uploaded as part of the Supplementary Materials [94], and the full code and auxiliary files are stored in GitHub: https://github.com/tahliapollock/Canine-tooth-biomechanics-BeamTheory-FinitieElementAnalysis and have been archived within the Zenodo repository: https://doi.org/10.5281/zenodo.7106870 [95]. Project files (.cae) for all finite-element models presented in the main text and in the Supplementary Materials can be found on Monash Bridges at https://bridges.monash.edu/projects/Canine_tooth_biomechanics_beam_theory_and_finite_element_analysis/140053. Input values for various loading conditions can be found in the Supplementary Materials.
